# A novel three-dimensional volumetric method to measure indirect decompression after percutaneous cement discoplasty

**DOI:** 10.1016/j.jot.2021.02.003

**Published:** 2021-04-01

**Authors:** Peter Endre Eltes, Laszlo Kiss, Ferenc Bereczki, Zsolt Szoverfi, Chloé Techens, Gabor Jakab, Benjamin Hajnal, Peter Pal Varga, Aron Lazary

**Affiliations:** a In Silico Biomechanics Laboratory, National Center for Spinal Disorders, Buda Health Center, Budapest, Hungary; b Department of Spine Surgery, Semmelweis University, Budapest, Hungary; c School of PhD Studies, Semmelweis University, Budapest, Hungary; d National Center for Spinal Disorders, Buda Health Center, Budapest, Hungary; e Biomechanics Lab, Department of Industrial Engineering, Alma Mater Studiorum, Universita di Bologna, Italy

**Keywords:** Indirect foraminal decompression, Computed tomography, Three-dimensional volumetric measurements, Minimally invasive spine surgery, Percutaneous cement discoplasty, Patient-specific simulation

## Abstract

**Purpose:**

Percutaneous cement discoplasty (PCD) is a minimally invasive surgical option to treat patients who suffer from the consequences of advanced disc degeneration. As the current two-dimensional methods can inappropriately measure the difference in the complex 3D anatomy of the spinal segment, our aim was to develop and apply a volumetric method to measure the geometrical change in the surgically treated segments.

**Methods:**

Prospective clinical and radiological data of 10 patients who underwent single- or multilevel PCD was collected. Pre- and postoperative CT scan-based 3D reconstructions were performed. The injected PMMA (Polymethylmethacrylate) induced lifting of the cranial vertebra and the following volumetric change was measured by subtraction of the geometry of the spinal canal from a pre- and postoperatively predefined cylinder. The associations of the PMMA geometry and the volumetric change of the spinal canal with clinical outcome were determined.

**Results:**

Change in the spinal canal volume (ΔV) due to the surgery proved to be significant (mean ΔV = 2266.5 ± 1172.2 mm^3^, n = 16; p = 0.0004). A significant, positive correlation was found between ΔV, the volume and the surface of the injected PMMA. A strong, significant association between pain intensity (low back and leg pain) and the magnitude of the volumetric increase of the spinal canal was shown (ρ = 0.772, p = 0.009 for LBP and ρ = 0.693, p = 0.026 for LP).

**Conclusion:**

The developed method is accurate, reproducible and applicable for the analysis of any other spinal surgical method. The volume and surface area of the injected PMMA have a predictive power on the extent of the indirect spinal canal decompression. The larger the ΔV the higher clinical benefit was achieved with the PCD procedure.

**The translational potential of this article:**

The developed method has the potential to be integrated into clinical software’s to evaluate the efficacy of different surgical procedures based on indirect decompression effect such as PCD, anterior lumbar interbody fusion (ALIF), lateral lumbar interbody fusion (LLIF), oblique lumbar interbody fusion (OLIF), extreme lateral interbody fusion (XLIF). The intraoperative use of the method will allow the surgeon to respond if the decompression does not reach the desired level.

## 1 Introduction

The intervertebral disc degeneration (IDD) leads to biomechanical and structural changes of the spine [1]. The degree of IDD can be defined by the MRI based Pfirrmann grading system [2], where the terminal disc degeneration (Pfirrmann V) is characterised by total disorganisation of the intervertebral tissue, the complete resorption of the nucleus pulposus and in many cases causing a vacuum phenomenon [[3], [4], [5]]. Discs act as transmitting units and shock absorbers, distributing the load of body weight and muscle activity through the spinal column [6]; therefore, degeneration related structural changes will lead to biomechanical dysfunctions [7], such as segmental instability. Decreasing disc height will generate continuously diminishing spinal canal dimensions which further deteriorate by dynamic changes of the neuroforamen due to movement. Lumbar spinal stenosis can lead to the development of chronic radiculopathy causing local and irradiating pain typically provoked by axial loading [8]. Surgical treatment possibilities of segmental instability in elderly patients are limited due to patients’ age, comorbidities and frailty. Therefore, minimally invasive surgical (MIS) procedures have become the preferred options. Percutaneous cement discoplasty (PCD) is a MIS procedure, where the vacuum space in the intervertebral disc is filled out with percutaneously injected PMMA (Polymethylmethacrylate), which provides a segmental stabilizing effect and indirect decompression of the neuronal elements due to the increase of the spinal canal dimensions. The technical details, the clinical effect and safety issues of the procedure have been previously published and the usage of the technique has also been supported by a prospective radiological study [[9], [10], [11], [12]].

Even though the spinal canal is a complex 3D geometry, the common description of its dimensions and the evaluation of the indirect decompression effect are based on 2D parameters. Measurements of disc height, foramen height/diameter, foramen cross-sectional area, central canal diameter, central canal cross-sectional area, or segmental lordosis angle therefore can possibly be biased [[13], [14], [15]]. However, the changes in the spinal canal (central canal and neuroforamen) dimensions have been quantified by only a few authors so far [16,17], because of the challenging methodological issues.

Navarro [16] in his study reveals the usefulness of advanced computational methods by demonstrating that volumetric analysis of the anatomical change can better predict the clinical outcome of Extreme Lateral Interbody Fusion (XLIF) compared to conventional 2D methods [18,19]. The developed computer algorithm by Gates and his colleagues [17] was used to investigate patient who underwent lateral retroperitoneal transpsoas interbody fusion (LIF) based on the pre and postoperative magnetic resonance images (MRI). To accurately calculate the changes of the spinal canal after a surgical procedure such as PCD, we aimed to develop a generalisable method based on patient-specific volumetric measurements, using 3D computational methods.

## 2 Methods

### 2.1 Clinical cohort and CT scan acquisition

We performed a retrospective analysis of prospectively collected data involving 10 consecutive patients (74 ​± ​7.7 years old), who underwent primary single or multilevel PCD at a tertiary care spine referral centre (Table 1). All patients suffered from low back pain and leg pain, due to advanced disc degeneration, and forward bending of the lumbar spine decreased the pain level, during physical examination.

**Table 1:** Clinical cohort.

n= ​10	Patient ID	Age (years)	Gender	Treated segment
	P01	83	M	L4-L5
	P02	59	F	L2-L3
				L3-L4
				L4-L5
	P03	67	M	L5-S1
	P04	78	F	L3-L4
	P05	79	M	L5-S1
	P06	76	F	L1-L2
	P07	75	F	L2-L3
				L3-L4
				L4-L5
	P08	66	M	L3-L4
				L4-L5
	P09	77	F	Th12-L1
				L1-L2
	P10	82	F	L1-L2

All presented operative procedures were performed by a single surgeon (GJ). Preoperative (preop), and postoperative (postop) 6-month follow-up results were collected and analysed using the Oswestry Disability Index (ODI) for spine specific function and with the visual analogue scale (VAS) for leg pain (LP) and low back pain (LBP). Patients participating in the study were informed and their written consent was obtained. The study was approved by the National Ethics Committee of Hungary, the National Institute of Pharmacy and Nutrition (reference number: OGYÉI/163–4/2019).

Quantitative Computed Tomography (QCT) scans were performed pre- and postoperatively, with a Hitachi Presto CT machine using an in-line calibration phantom, and a protocol previously defined in the MySpine study (ICT-2009.5.3 VPH, Project ID: 269909) with an intensity of 225 ​mA and voltage of 120 ​kV [20,21]. Images were reconstructed with a voxel size of 0.6x0.6x0.6 ​mm^3^. Based on the QCT images, Hounsfield Units can be converted into bone mineral density (BMD) equivalent values, which are necessary for creating finite element (FE) models. In this study the QCT images were used as conventional CT images without any conversion. The data were exported from the hospital’s PACS into DICOM file format. To comply with the ethical approval and the patient data protection, anonymization of the DICOM data was performed using the freely available Clinical Trial Processor software (Radiological Society of North America, https://www.rsna.org/ctp.aspx) [22].

### 2.2 Definition of pre- and postop motion segments’ 3D geometry

**Figure 1:**
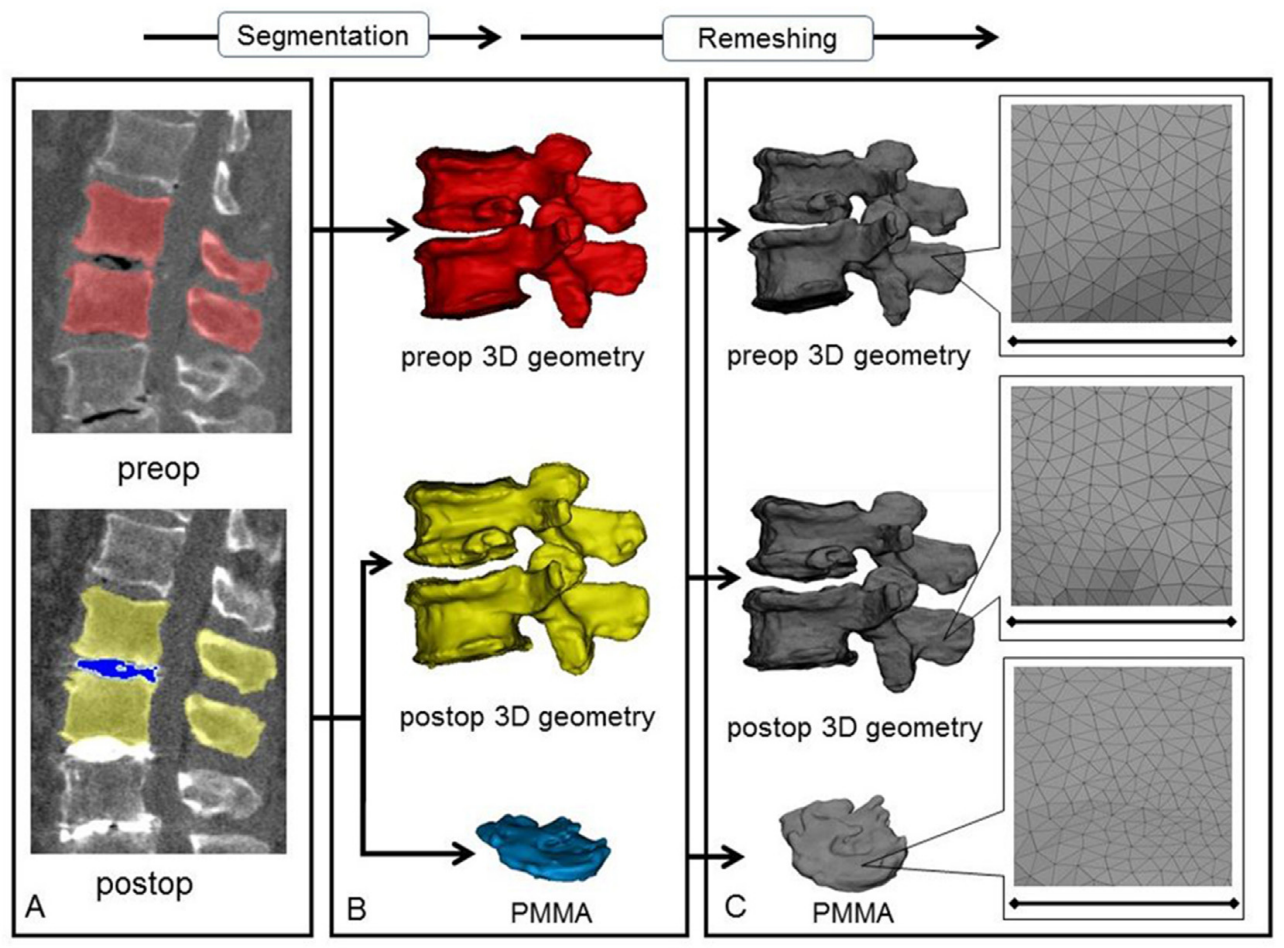
3D geometry definition of pre- and postoperative motion segment geometries and of the injected PMMA geometry. **A** During the segmentation process the bone volume is first separated from the surrounding soft tissue by thresholding of the Hounsfield units’ levels of the 2D CT images (sagittal view). The resulting red, yellow and blue masks represent the volumes of the pre- and postoperative vertebrae, and the PMMA respectively. **B** From the mask, a triangulated surface mesh is generated, and smoothing is applied (iteration: 6, smooth factor: 0.7, with shrinkage compensation). **C** Uniform remeshing was applied (target triangle edge length: 0.6 ​mm, sharp edge preservation, sharp edge angle: 60°). Scale bar length is 5 ​mm.

In order to establish the 3D vertebral geometry of the pre- and postoperative motion segments and the injected polymethyl methacrylate (PMMA) geometry, a segmentation process was performed on the 2D CT images [23]. 16 motion segments were treated and analysed in a cohort of 10 patients. The thresholding algorithm and manual segmentation tools (erase, paint, fill etc.) in Mimics® image analysis software (Mimics Research, Mimics Innovation Suite v21.0, Materialise, Leuven, Belgium) were used (Fig. 1). During the segmentation process the bone volume was first separated from the surrounding soft tissue by thresholding of the Hounsfield units’ levels. The developed masks (group of voxels) were homogenously filled by preserving the outer contour of the geometrical border in 2D. From the mask, a triangulated surface mesh was automatically generated. On the 3D geometries surface smoothing was applied (iteration: 6, smooth factor: 0,7, with shrinkage compensation). Furthermore, uniform remeshing process was carried out (target triangle edge length 0.6 ​mm, sharp edge preservation, sharp edge angle 60°) for all vertebrae and PMMA geometries. To evaluate the accuracy of the segmentation process, we calculated the Dice Similarity Index (DSI) [24], [25]. The DSI quantifies the relative volume overlap between two segmentation procedures as follows: $DSI=\frac{2\cdot V\left(I₁\cap I₂\right)}{V\left(I₁\right)+V\left(I₂\right)},$ *V* is the volume of the voxels inside the binary mask (number of voxels multiplied with the voxel size; in mm^3^), and I_*1*_ and I_*2*_ are the binary masks from two segmentation processes (performed by two investigators (Ii_1_ and I_2_). The DSI values range between 0 ​- ​1, where 1 denotes a perfect match. Accuracy of the vertebral geometry segmentation was evaluated by a random (Microsoft Office Professional Plus 2016, Excel, RANDBETWEEN function) selection of 6 preoperative and 6 postoperative vertebral geometries. All 12 vertebras were segmented by the two investigators (I_1_ and I_2_) and the two segmentations were compared after then the DSI was defined. The PMMA segmentation evaluation was done by repeating all the 16 measurements by I_2_ and then the DSI was calculated.

### 2.3 Alignment of the motion segments’ geometry

**Figure 2:**
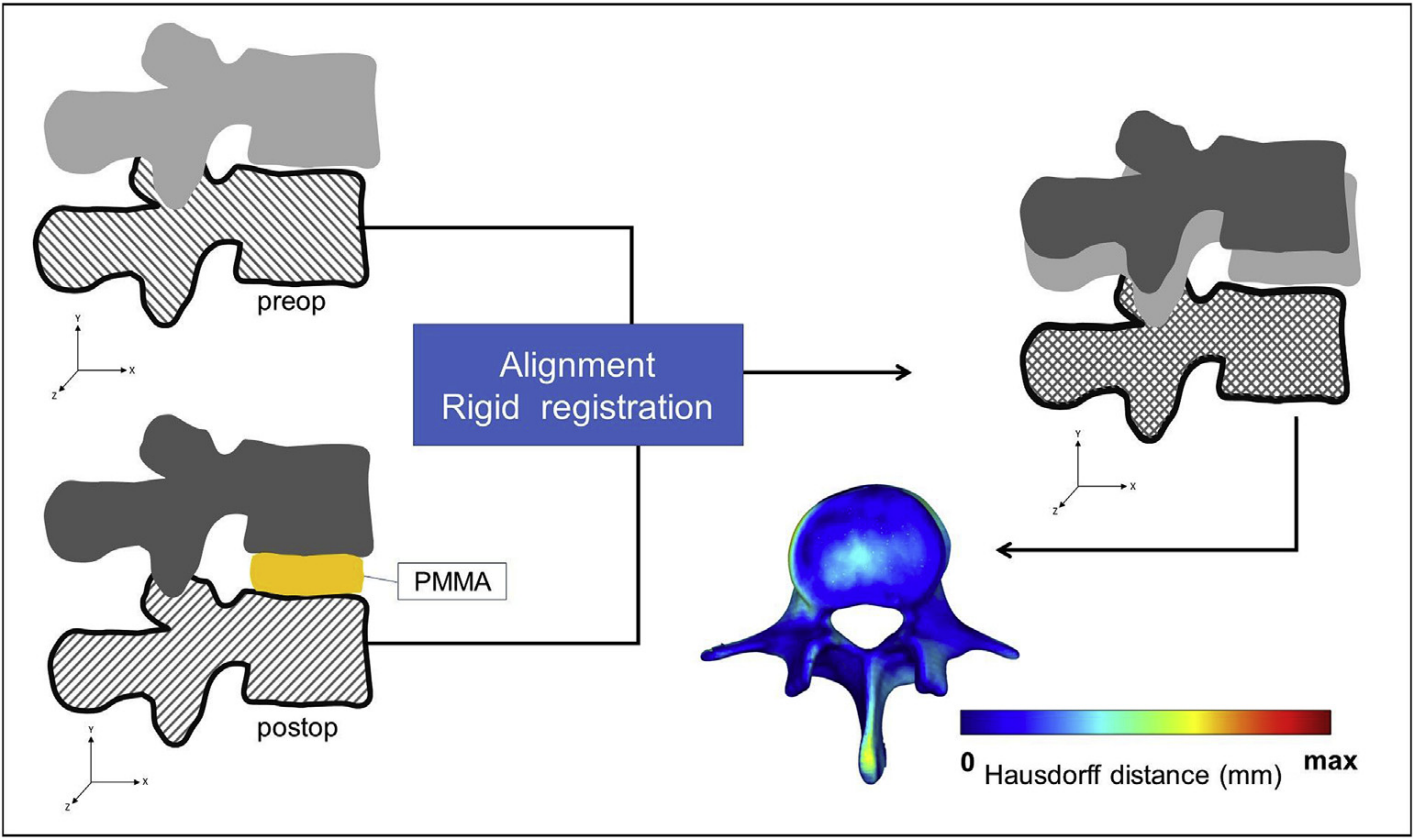
Alignment of the preoperative motion segment vertebral geometry to the postoperative geometry. The alignment of the caudal vertebra was performed by using control points (as shown in Online Resource 1.) and rigid surface registration algorithms. The process created a common coordinate system for the pre- and postoperative motion segments with nearly identical boundaries for the caudal vertebras. The Hausdorff Distance was used as a quality measure for the alignment process at the caudal vertebra.

To detect the PCD induced postoperative changes in the motion segment, the pre- and postoperative vertebral geometries were aligned in the same coordinate system. For this, preoperative 3D datasets were transposed into the same coordinate system as the postoperative data. Pre- and postop caudal vertebral surface mesh models of the treated motion segments were used as reference geometry (Fig. 2) and a control points based rigid registration algorithm was used via Mimics® software. The 18 control points corresponded to easily identifiable anatomical landmarks at the caudal vertebra and the sacrum (Online Resource 1). To evaluate the accuracy of the registration and alignment procedure, the Hausdorff Distance (HD) was measured with the MeshLab1.3.2 software [26] (http://www.meshlab.net) Metro tool [27] (Fig. 2) at the level of the aligned caudal pre- and postoperative vertebras. The HD represents the maximum distance between two points (triangle vertex) of two sets, both from corresponding sections of the meshes (i.e.: the HD is expected to be equal to zero in case of a perfect alignment of absolute symmetrical geometries, whereas values >0 provide the actual distance between the two surfaces). The HD values were calculated at the vertices of the triangulated surface meshes as follows: $h\left(A,B\right)=max_{a\varepsilon A}\left\{min_{b\varepsilon B}\left\{d\left(a,b\right)\right\}\right\}$ ; where *A* is the postop mesh; *B* is the preoperative reference mesh; *a* and *b* are points of sets *A* and *B* respectively, and *d(a, b)* is the Euclidian metric between these points. The alignment measurements of the pre- and postop motion segments were performed by two investigators (I_1_, I_2_) and two aligned datasets were created with 16-16 motion segments each. The HD measurements were performed for all the 16 registered motion segments by both investigators.

### 2.4 Measurement of the neuroforaminal 3D geometry

**Figure 3:**
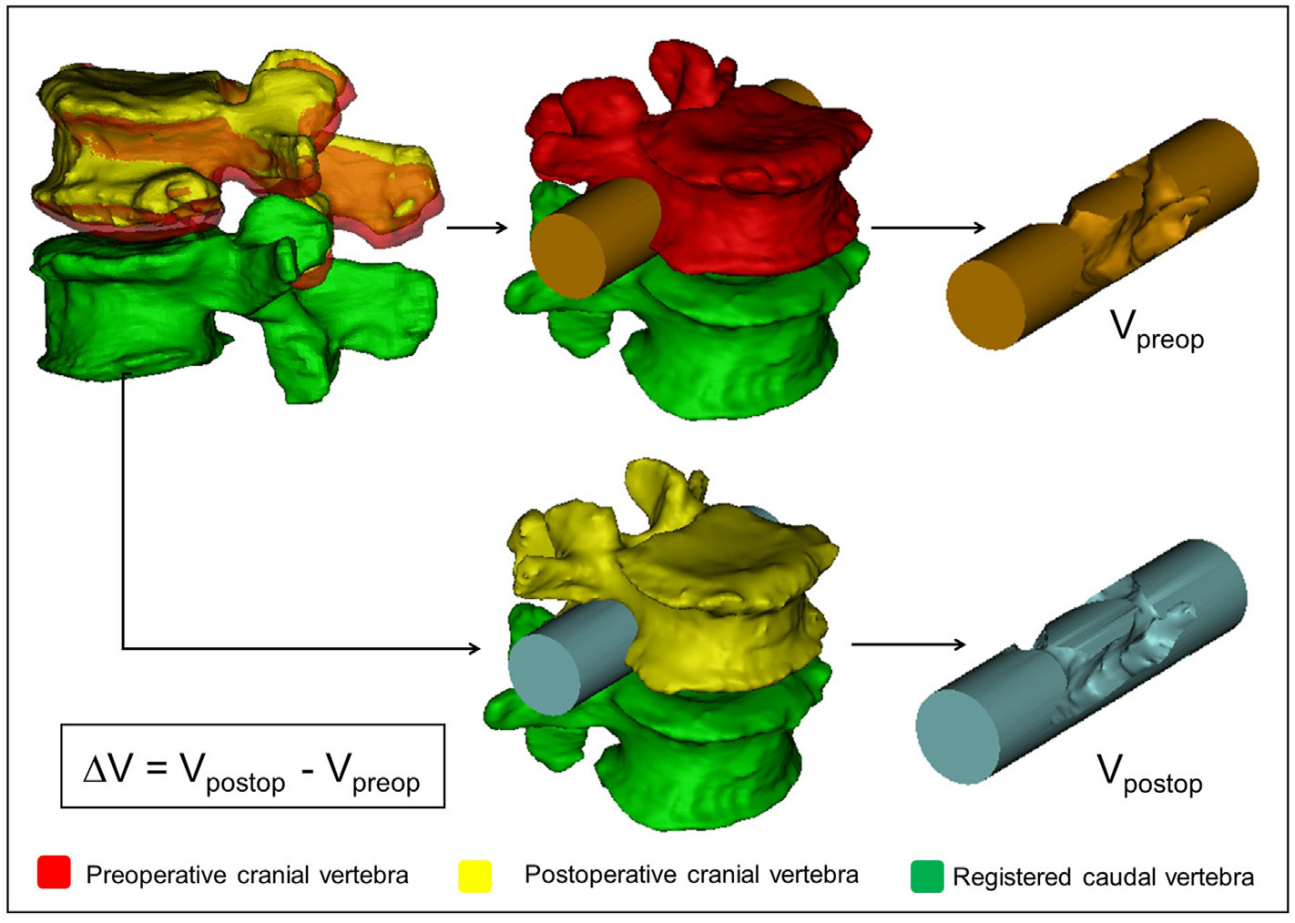
Measurement of the PCD induced changes in the neuroforaminal geometry. After alignment, the pre- and postoperative motion segments shared a common caudal vertebra. The cranial vertebra geometrical position has changed due to the lifting effect of the PMMA. Two identical cylinders were introduced in the neuroforaminal and central canal regions of the pre- and postoperative motion segments. V_preop_ and V_postop_ represent the subtraction of the overlapping vertebral geometry from the initial cylinder geometry. The indirect decompression effect of the PCD is defined as ΔV (ΔV = V_postop_ - V_preop_).

After the alignment, change in spinal canal geometry was defined by using a measurement cylinder created using Mimics® software and inserted in the virtual coronal axis of the neuroforamina (coronal plane). Its length was defined at 90 ​mm, while the radius of the cylinder was set by the investigators uniquely in each patient and segment (Table 2) in a way to fill the neuroforamina’s volumes and the central canal in pre- and postoperative 3D geometries of the motion segments (Fig. 3). The overlapping volumes between the cylinder and the motion segment 3D geometry were subtracted (Boolean operation/Minus). The change in the subtracted cylinder volumes represents the spinal canal dimension V_preop_ ​= ​V(Cylinder ∩ Preop motion segment), and V_postop_ ​= ​V(Cylinder ∩ Postop motion segment). Change in the subtracted cylinder volumes represents the indirect decompression effect of the surgical procedure and it is defined as ΔV (ΔV = V_postop_ - V_preop_) and is shown in Fig. 3. To determine the repeatability and accuracy of the measurement method, intra- and interrater reliability analysis was conducted of the two investigators (I_1_,I_2_) at two different time points (T_1_, T_2_).

**Table 2:** Mean volumes and changes based on the measurements of I_1,_ I_2,_ I_3_, I_4_ at T_1_ and T_2_ time points ((I_1_T_1_+I_1_T_2_+I_2_T_1_+I_2_T_2_ I_3_T_1_+I_3_T_2_+I_4_T_1_+I_4_T_2_)/8). SD: Standard Deviation; CV: Coefficient of Variation.

Patient ID	Treated segment	Cylinder height (mm)	Cylinder radius (mm)	Average subtraction (Preop. mm³)	Avearage subtraction (Postop. mm³)	Avearage Δvolume (mm³)	SD Δ volume (mm³)	CV Δ volume (mm³)
P01	L4-L5	90	11	23173.72	26760.96	3587.24	216.62	0.06
P02	L2-L3	90	10	22259.45	24031.61	1772.16	78.81	0.04
	L3-L4	90	11	25816.38	28998.01	3181.62	91.77	0.03
	L4-L5	90	10	18533.00	22001.31	3468.31	79.20	0.02
P03	L5-S1	90	10	10815.84	14296.92	3481.09	140.95	0.04
P04	L3-L4	90	12	31104.05	33326.11	2222.06	106.59	0.05
P05	L5-S1	90	11	14625.24	18575.94	3950.70	87.83	0.02
P06	L1-L2	90	10	21080.61	22532.95	1452.34	59.83	0.04
P07	L2-L3	90	10	21513.30	22970.16	1456.87	64.30	0.04
	L3-L4	90	10	20396.61	22698.79	2302.18	167.90	0.07
	L4-L5	90	10	18468.15	21559.50	3091.35	114.93	0.04
P08	L3-L4	90	11	24068.52	25273.62	1205.09	267.77	0.22
	L4-L5	90	12	26523.69	30013.21	3489.52	305.05	0.09
P09	Th12-L1	90	10	22695.25	23792.51	1097.26	145.93	0.13
	L1-L2	90	10	22351.12	22898.23	547.11	51.89	0.09
P10	L1-L2	90	10	23986.02	24403.36	417.34	49.25	0.12

### 2.5 PMMA geometry visualisation and thickness measurement

The 3D geometry of the intervertebral PMMA for the 16 treated motion segments were defined during the segmentation process by a uniformly remeshed triangulated surface mesh (Fig. 1). The surface mesh defines the geometry and determines the surface and volume of the investigated object. Thickness measurement was performed and visualised by using contour plots by applying the 3-matic® software (Mimics Innovation Suite v21.0, Materialise, Leuven, Belgium). Thickness was defined at the level of every triangle element of the surface mesh as the perpendicular distance from the element midpoint to the other wall (surface) of the geometry.

### 2.6 Statistical analysis

All statistical tests were performed with SPSS Statistics 25.0 (IBM Corp., Armonk, NY, USA) and due to small sample size nonparametric tests were applied. The HD measurements cumulative probability plots (Online Resource 2) were created with SigmaPlot 12 (SSI, San Jose, California, United States). Interrater (I_1_ vs I_2_ vs I_3_ vs I_4_) reliability was determined by Intraclass Correlation Coefficient (ICC) estimates and their 95% confident intervals (CI) were calculated based on a mean-rating (k ​= ​4), absolute-agreement, 2-way mixed-effects model. Intrarater (I_1_T_1_ vs I_1_T_2_, I_2_T_1_ vs I_2_T_2_, I_3_T_1_ vs I_3_T_2_, I_4_T_1_ vs I_4_T_2_) reliability was determined by ICC estimates and their 95% confident intervals were calculated based on a single measurement, absolute-agreement, 2-way mixed-effects model. The statistical difference in the change between the pre- and postoperative spinal canal volume, ODI, LP and LBP was assessed by Paired Sample Wilcoxon signed ranked test. The relationships between the PMMA and the mean volumetric change (ΔV); PMMA surface and ΔV; PMMA surface-volume ratio and ΔV were defined using the Spearman’s rank correlation. The associations between the ΔODI (preop – postop) and the ΔV; ΔLP (preop – postop) and the ΔV, ΔLBP (preop – postop) and the ΔV were investigated by the Spearman’s rank correlation. P-values less than 0.05 were considered significant. Power analysis was used to determine the sample size for the Wilcoxon signed ranked tests and Spearman’s correlations. by applying G-POWER [28,29] using an alpha of 0.05. Our sample size (N ​= ​16 for treated segment, N ​= ​10 for patient) provided a more than 0.8 power to the study, with a large effect size (|ρ| ​= ​0.72 for the pre- and postoperative spinal canal change, |ρ| ​= ​0.6 for the relationship between (ΔV) and PMMA surface, volume, and surface-to-volume ratio, |ρ| ​= ​0.71 for the relationship between ΔV and ΔODI, ΔLP, ΔLBP).

## 3 Results

### 3.1 Evaluation of the segmentation procedure

**Figure 4:**
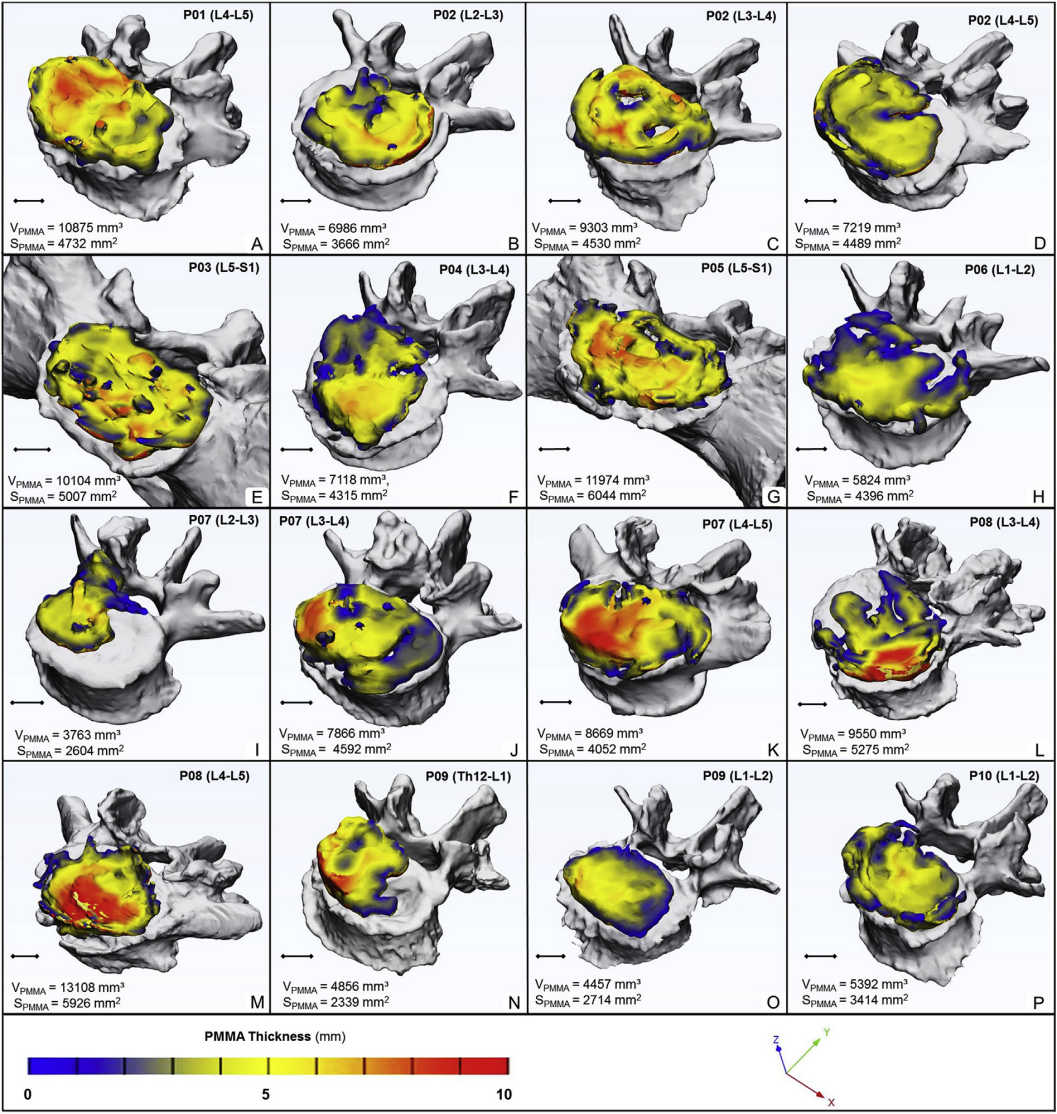
Visualization and thickness measurement of the PMMA geometry injected during PCD. **A-P** the PMMA geometry distribution over the caudal vertebral endplate of the investigated motion segment (the xyz coordinate system defines the view). The average volume is 7941.59 ​± ​2749.82 ​mm³, and surface is 4256.02 ​± ​1094.20 ​mm^2^. Thickness is represented by the colorbar (Blue/Green/Red), scale 0–10 ​mm (A–P).

To evaluate the accuracy of our segmentation process we used the DSI for 6 randomly selected geometries (Online Resource 3). The obtained DSI values for both pre- and postoperative geometries were 0.96 ​± ​0.02 and 0.90 ​± ​0.07, respectively (n ​= ​6) and showed negligible variance, thus indicating a high accuracy of our segmentation method for all segmented geometries [30]. Next, to assess the injected PMMA cement geometries we first evaluated the PMMA geometry distribution over the caudal vertebra endplate of the motion segments visually "in the same 3D view (Fig. 4). Because the degenerative processes are not only age dependent, but also rely on the musculoskeletal status of each patient, the variations of intervertebral disc degeneration will be widely different. Accordingly, we found that the injected volumes are arranged patient-specifically to widely differing 3D shapes (Fig. 4). Because of this large variance, is unlikely for a random subset to be representative of the whole"; therefore, we chose to validate the segmentation process on all injected PMMA volumes instead. We calculated the DSI as above described for the 16 segmented geometries (Online Resource 4). Again, the DSI values were very high for all segmented geometries (mean: 0.93 ​± ​0.035, n ​= ​16) demonstrating the precision of our segmentation method also in the case of the injected PMMA geometries.

### 3.2 Motion segments alignment evaluation

Having confirmed the precision of our segmentation process, we next evaluated the accuracy of the alignment of the pre-and postoperative motion segments by measuring HD values. The same processes were performed by two investigators. The HD values represent the maximal distance between two corresponding points (vertex) of the respective registered surface meshes. We obtained a mean HD value of 0.43 ​± ​0.19 ​mm for the first investigator (I_1_), and 0.54 ​± ​0.16 ​mm for the second investigator (I_2_) indicating adequate fitting [31] (Online Resource 5). To obtain a detailed view on the precision of our alignment, we created cumulative probability plots for the measured HD values for both investigators. We found that the maximal distance between the registered pre- and postoperative 3D geometries was almost always (90%) smaller than 2 ​mm, and ~70% of the values were smaller than 1 ​mm (Online Resource 2). These measurement results confirm the accuracy of registration and alignment methods. Consequently, the calculation of volumetric changes of the spinal canal, are expected to be similarly precise.

### 3.3 PCD induced indirect volumetric decompression

**Figure 5:**
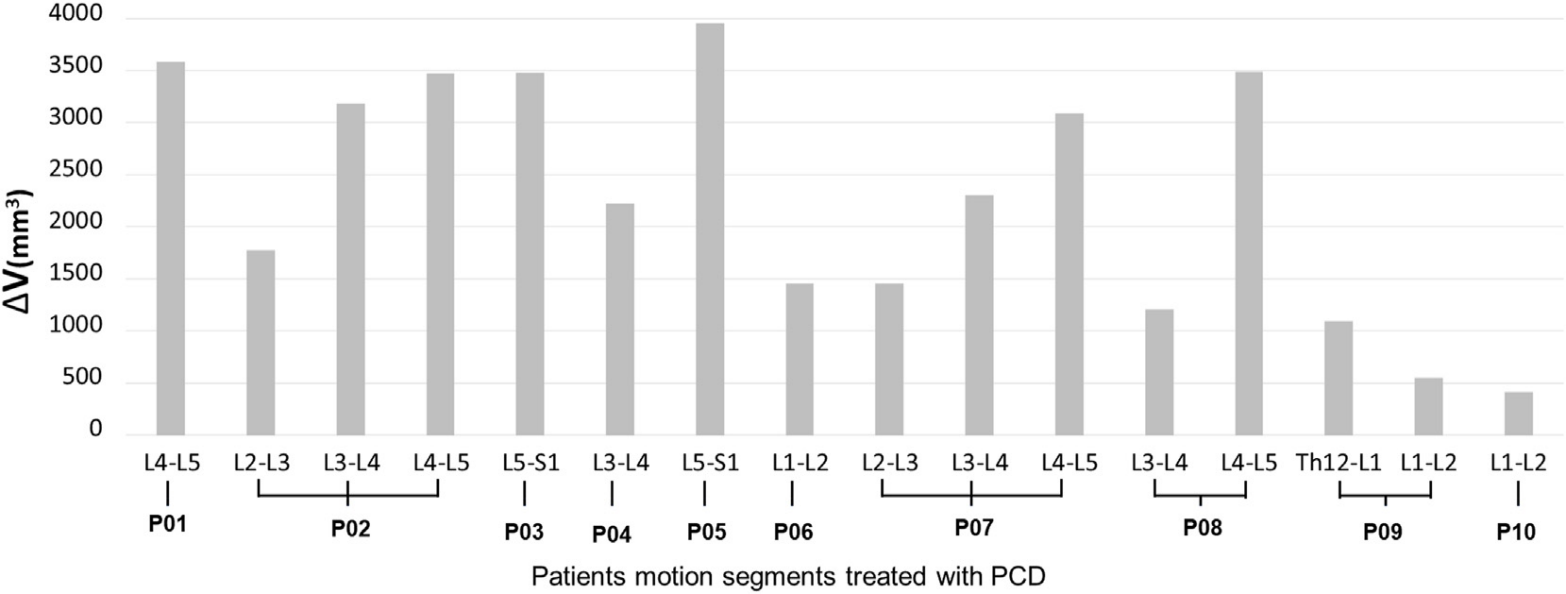
Distribution of PCD induced mean volumetric change (ΔV) of the spinal canal. An average of 2295.14 ​± ​1181.42 ​mm³ volumetric increase was measured (16 PCD treated segments, n ​= ​10 patients.). We found a significant geometrical change between the mean pre- and postoperative spinal canal volume (V_postop_ vs V_preop_, p ​= ​0.0004).

To test the accuracy and reproducibility of these measurements, we involved four investigators (I_1_ vs I_2_ vs I_3_ vs I_4_) who performed the same procedures at two time points (T_1_ vs T_2_). We found that intrarater reliability for I_1_T_1_ vs I_1_T_2_ was ICC ​= ​0.999 (CI 95%, Lower Bound ​= ​0.998, Upper Bound ​= ​1); for I_2_T_1_ vs I_2_T_2_ ICC ​= ​0.994 (CI 95%, Lower Bound ​= ​0.984, Upper Bound ​= ​0.998); for I_3_T_1_ vs I_3_T_2_ ICC ​= ​0.997 (CI 95%, Lower Bound ​= ​0.990, Upper Bound ​= ​0.999); for I_4_T_1_ vs I_4_T_2_ ICC ​= ​0.996 (CI 95%, Lower Bound ​= ​0.987, Upper Bound ​= ​0.999) The interrater reliability for I_1_ (mean T_1_, T_2_) vs I_2_ (mean T_1_, T_2_) vs I_3_ (mean T_1_, T_2_) vs I_4_ (mean T_1_, T_2_) was found to be ICC ​= ​0.997 (CI 95%, Lower Bound ​= ​0.992, Upper Bound ​= ​0.999). Results of the reliability measurement indicate a high accuracy and reproducibility of the volumetric change measurements (Online Resource 6,7,8,9). The actual volumetric change (ΔV) of the spinal canal in the PCD-treated motion segments was determined as ((I_1_T_1_+I_1_T_2_+I_2_T_1_+I_2_T_2_+I_3_T_1_+I_3_T_2_+I_4_T_1_+I_4_T_2_)/8) (Table 2). The distribution of the actual volumetric change is presented in Fig. 5. The volumetric changes are widely differing, similarly to the shape of the injected volumes (Fig. 4). The observed geometrical change (mean ​= ​2295.14 ​mm^3^, SD ​= ​1181.42, n ​= ​16) between the pre- and postoperative measurement cylinder volumes demonstrates a significant difference (V_postop_ vs V_preop_, p<0.001 Fig. 5).

### 3.4 Corelation between PMMA geometry and the volumetric change (ΔV) of the spinal canal

**Figure 6:**
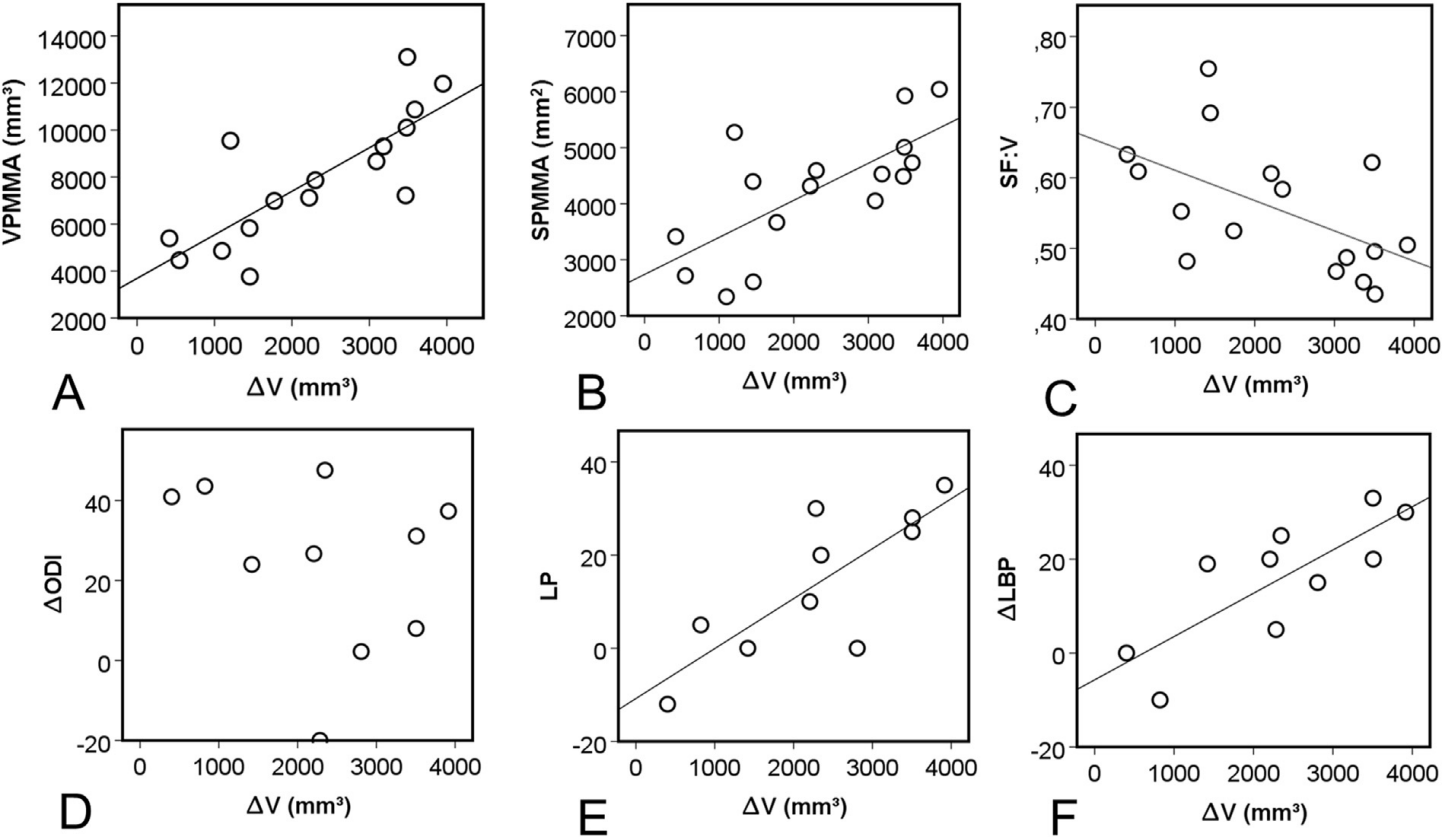
Association of the mean volumetric change (ΔV) of the spinal canal induced by the PCD with the PMMA volume, surface, surface-to-volume ratio (SF:V) and with the clinical outcome (ODI,LP,LBP). A, B We found significant, positive correlation between the PMMA volume, PMMA surface and ΔV (ρ ​= ​0.762, p ​= ​0.001 and ρ ​= ​0.668, p ​= ​0.005). C The correlation between SF:V and ΔV although moderate, was found to be significantly negative (ρ ​= ​−0.535, p ​= ​0.033). D The negative, weak correlation was found not to be significant between the change of the ODI and ΔV (ρ ​= ​−0.321, p ​= ​0.365). E, F positive, significant and strong correlation was found between the ΔLP, ΔLBP and ΔV (ρ ​= ​0.772, p ​= ​0.009, and ρ ​= ​0.693, p ​= ​0.026 respectively). For D, E, F a patient averaged ΔV was used for patients who underwent multiple segment PCD.

A significant, strong, positive correlation was found between the volume of the injected PMMA volume and the ΔV of the spinal canal (ρ ​= ​0.821, p ​= ​0.001) (Fig. 6). The surface area of the discoplasty showed a significant and strong, positive correlation with the volumetric change of the spinal canal (ρ ​= ​0.729, p ​= ​0.001). A significant and moderate [32], negative correlation was found between the PMMA surface-to-volume ratio (SF:V) and the volumetric change of the spinal canal (ρ ​= ​−0.565, p ​= ​0.023).

### 3.5 Clinical outcome

ODI, LBP and LP significantly decreased 6 months after the PCD procedure (p ​= ​0.013; p ​= ​0.036; p ​= ​0.015; respectively, Online Resource 10) and the magnitude of change was more than the minimal clinically important difference (MCID) (ODI: 24 points, 16 points in LBP, and 14 points in LP respectively). Strong, significant correlations between the improvement of pain and spinal canal ΔV were found (ρ ​= ​0.754, p ​= ​0.012 for LBP and ρ ​= ​0.736, p ​= ​0.015 for LP, respectively) (Fig. 6). Correlation between ODI and ΔV was weak and not significant (ρ ​= ​−0.212, p ​= ​0.556).

## 4 Discussion

PCD is a MIS option to reduce low back pain caused by severe disc degeneration especially in the elderly. The actual volumetric change and decompressive effect of the procedure was not quantified previously because of the lack of an appropriate 3D measurement method. PCD is not the only surgical procedure, where the clinical effect is at least partly related to the indirect decompression of the spinal canal. The highly accurate method described in the present study provides an exact and feasible option for the quantitative analysis of 3D changes of the spinal canal after the different fusion techniques. The newly developed approach precisely assesses the effect of different surgical techniques and by that, it provides a novel possibility for evidence-based comparison. Our method compared to Navaro’s [16] and Gates [17] approach uses the caudal vertebra surface, as a common coordinate system for pre and postoperative comparative measurement’s, allowing complex visualizations and data collection about the treated motion segment. Gates’s publication lacks some important methodological details making the algorithm difficult to reproduce. In our method there is the possibility to fully automate the spine segmentation, registration and cylinder insertion with the aid of artificial intelligence (AI) algorithms [33,34]. This is a practical advantage of our method compared to Navaro’s approach.

We found that PCD leads to a significant increase of the spinal canal dimensions providing a clinically important indirect decompression effect. The injected PMMA distribution in the intervertebral space influences the decompression volume, with higher volume, larger surface and lower surface-volume ratio a greater decompression can be achieved. The PCD procedure improves the disability and pain of the patients. At 6-month follow-up we measured a 24 points improvement in ODI, 16 points in LBP, and 14 points in LP, which is more than the minimal clinically important change in ODI and VAS [35]. The pain relief effect of the procedure significantly correlated with the measured volumetric change of the spinal canal (ie. the indirect decompression). This also indicates a volume dependent improvement of patient symptoms, with a higher injected PMMA volume resulting in better treatment outcome. However, disability was not associated with volumetric change, indicating that the function of the patient is a multidimensional feature also influenced by the patient’s lifestyle, general health status and other comorbidities.

The present study provides scientific evidence on the indirect decompression effect of the PCD procedure with the application of a novel computational method, however, there are some possible limitations of the study and the explanation of our results. Despite the fact, that the measurement and simulation method showed high accuracy and repeatability, we cannot exclude that more complex local anatomical variations can influence the application of the method. The external validation of the published measurement method even in another patient group (different type of surgery) would be also desirable. Change of clinical symptoms (ie. pain) and especially disability have multifactorial characteristics, therefore we could not determine the direct effect of the indirect decompression. PCD is also expected to increase the biomechanical stability of the motion segment [36], which can also relieve pain and improve function. Further biomechanical, computational research as well as large, multicenter cohort studies are required to clarify these open questions.

## 5 Conclusion

Patient specific computational methods provide accurate information about the unique and complex geometrical/anatomical relations caused by intervertebral disc degeneration. In the present study, the 3D geometrical change of the spinal canal and the indirect decompression effect of PCD, a minimally invasive surgical procedure, was investigated with a new computational 3D volumetric measurement method.Significant associations have been explored between indirect decompression and clinical improvement. Due to its relative simplicity we suggest the application of our measurement method for the scientific and clinical analysis of other surgical procedures based on indirect decompression effect such as: ALIF, LLIF, OLIF, XLIF.

## Ethical approval

All procedures performed in studies involving human participants were in accordance with the ethical standards of the institutional and/or national research committee and with the 1964 Declaration of Helsinki and its later amendments or comparable ethical standards.

## Informed consent

Informed consent was obtained from all individual participants included in the study.

## Human and animal rights

This article does not contain any studies involving animals performed by any of the authors.

## Declaration of competing interest

The authors declare that they have no conflict of interest.
