# Supplementary material for: A novel three-dimensional volumetric method to measure indirect decompression after percutaneous cement discoplasty
Source: J Orthop Translat. 2021 Apr 1;28:131–9. doi: 10.1016/j.jot.2021.02.003 (PMC8050383; doi:10.1016/j.jot.2021.02.003)
Supplement: Multimedia component 1 [file mmc1.pdf]

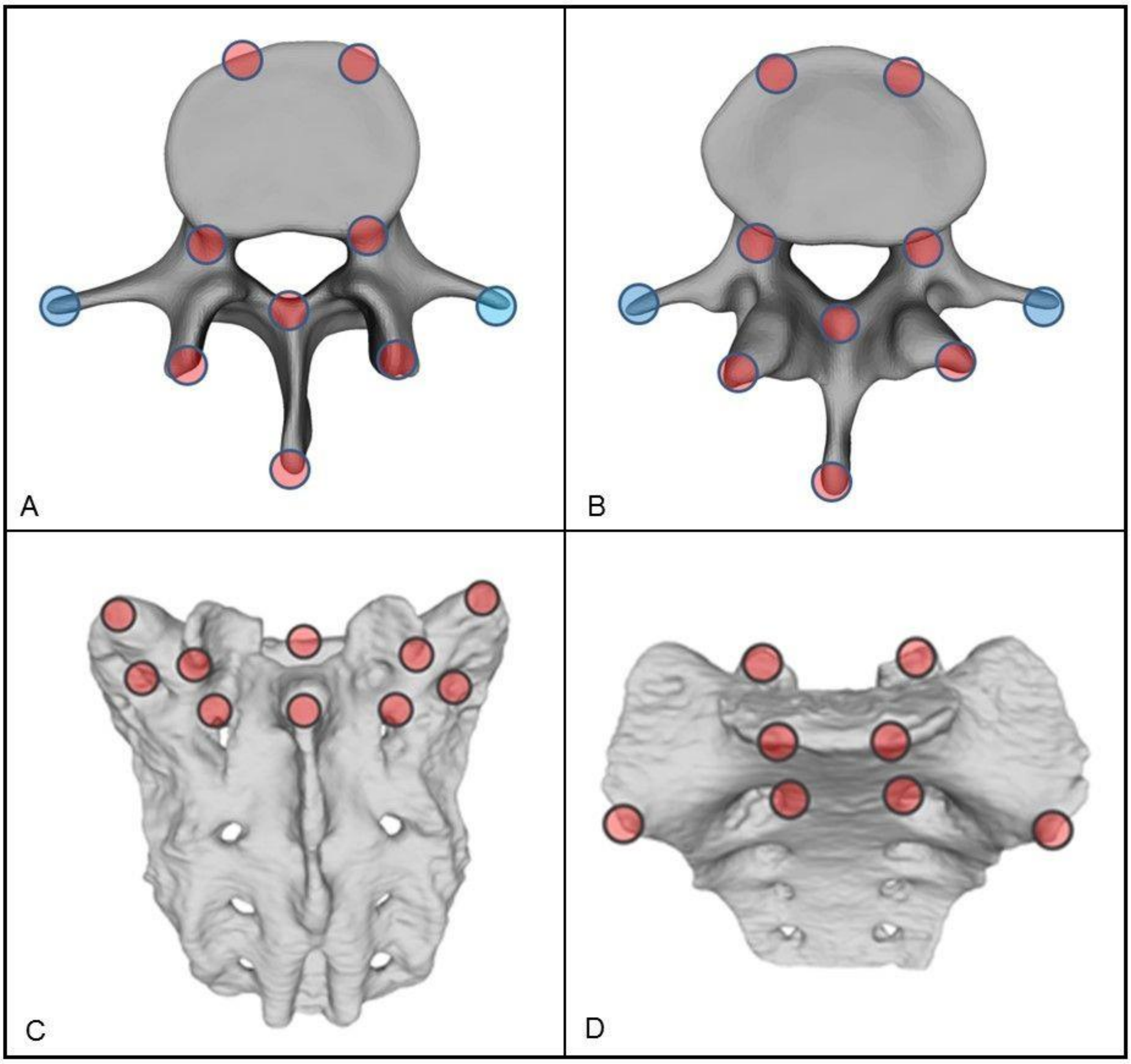

Online Resource 1. Selection of control points for rigid surface registration

Ten control points were selected from the superior (**A**) and eight from the inferior (**B**) reference regions of the vertebra and from the aligned geometry, respectively. For the sacrum ten control points were selected from the superior-dorsal (**C**) and eight from the superior-ventral (**D**) regions of the reference and from the aligned geometry, respectively. The filled red circles represent the selection areas of the registration points, the filled blue circles represent common selection areas for two different regions.
