# Supplementary material for: A novel three-dimensional volumetric method to measure indirect decompression after percutaneous cement discoplasty
Source: J Orthop Translat. 2021 Apr 1;28:131–9. doi: 10.1016/j.jot.2021.02.003 (PMC8050383; doi:10.1016/j.jot.2021.02.003)
Supplement: Multimedia component 2 [file mmc2.pdf]

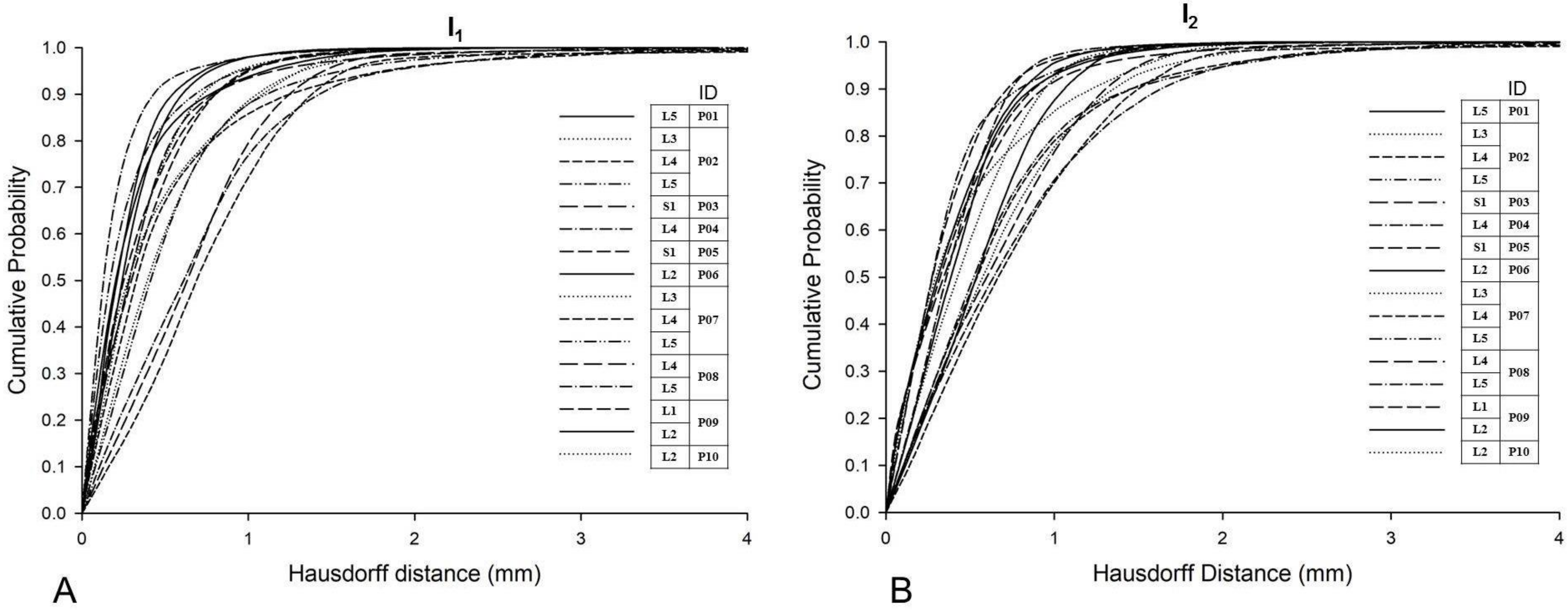

Online Resource 2. **Distribution of HD values between the surface meshes of the registered vertebras.**

A-B Cumulative probability plots of HD values for preoperative and postoperative caudal vertebra models of the treated motion segments. Approximately 90% percent of HD values are <2 mm and ~80% < 1 mm for all I_1 registrations, and ~70% < 1 mm for all I_2 registrations (I, investigator).
