# Supplementary material for: A novel three-dimensional volumetric method to measure indirect decompression after percutaneous cement discoplasty
Source: J Orthop Translat. 2021 Apr 1;28:131–9. doi: 10.1016/j.jot.2021.02.003 (PMC8050383; doi:10.1016/j.jot.2021.02.003)
Supplement: Multimedia component 3 [file mmc3.pdf]

	preop			postop	
Patient ID	***Vertebra***	***DSI***	***Patient ID***	***Vertebra***	***DSI***
**P01**	L4	0.97	P01	L5	0.96
**P02**	L3	0.98	P02	L4	0.83
**P04**	L4	0.96	P05	L5	0.96
**P06**	L2	0.99	P07	L4	0.91
**P08**	L3	0.96	P08	L5	0.81
**P09**	L1	0.93	P09	T12	0.96
	**mean DSI** 0.96 ±0.02			**mean DSI** 0.90 ±0.07	

Online Resource 3.
Evaluation of the accuracy of the segmentation process by two investigators (I_1, I_2) via Dice Similarity Index (DSI)
