# Supplementary material for: A novel three-dimensional volumetric method to measure indirect decompression after percutaneous cement discoplasty
Source: J Orthop Translat. 2021 Apr 1;28:131–9. doi: 10.1016/j.jot.2021.02.003 (PMC8050383; doi:10.1016/j.jot.2021.02.003)
Supplement: Multimedia component 4 [file mmc4.pdf]

DSI PMMA geometry

Patient ID	*Treated Segment*	*DSI*
**P01**	L4-L5	0.90
	L2-L3	0.94
**P02**	L3-L4	0.91
	L4-L5	0.92
**P03**	L5-S1	0.89
**P04**	L3-L4	0.96
**P05**	L5-S1	0.95
**P06**	L1-L2	0.85
	L2-L3	0.97
**P07**	L3-L4	0.97
	L4-L5	0.90
**P08**	L3-L4	0.95
	L4-L5	0.96
**P09**	Th12-L1	0.94
	L1-L2	0.91
**P10**	L1-L2	0.96

mean DSI 0.93 ±0.035

Online Resource 4.
Dice Similarity Index (DSI) values for the segmented PMMA geometries by two investigators (I_1, I_2)
