# Supplementary material for: A novel three-dimensional volumetric method to measure indirect decompression after percutaneous cement discoplasty
Source: J Orthop Translat. 2021 Apr 1;28:131–9. doi: 10.1016/j.jot.2021.02.003 (PMC8050383; doi:10.1016/j.jot.2021.02.003)
Supplement: Multimedia component 5 [file mmc5.pdf]

			I_1 HD (mm)				I_2 HD (mm)			
Patient ID	***Treated segment***	***Registred vertebra***	***Min***	***Max***	***Mean***	***RMS***	***Min***	***Max***	***Mean***	***RMS***
**P01**	L4-L5	L5	0	3.068747	0.3198	0.4824	0	3.1518	0.4488	0.5743
	L2-L3	L3	0	3.9796	0.3592	0.4844	0	3.3946	0.4835	0.6036
**P02**	L3-L4	L4	0	7.6414	0.7871	1.031	0	7.6193	0.7934	1.0651
	L4-L5	L5	0	3.8135	0.538	0.7369	0	4.3207	0.6859	0.9356
**P03**	L5-S1	S1	0	4.5732	0.387	0.5869	0	4.5271	0.4506	0.6505
**P04**	L3-L4	L4	0	2.8971	0.0209	0.3218	0	2.9924	0.4034	0.499
**P05**	L5-S1	S1	0	3.848	0.3749	0.5076	0	4.1867	0.4076	0.5564
**P06**	L1-L2	L2	0	2.7531	0.2982	0.3913	0	2.5098	0.5695	0.6759
	L2-L3	L3	0	5.7976	0.5107	0.7063	0	5.1411	0.6803	0.8733
**P07**	L3-L4	L4	0	5.8169	0.5412	0.8603	0	6.0648	0.7059	0.9826
	L4-L5	L5	0	3.0732	0.2819	0.4379	0	3.1539	0.3648	0.5092
**P08**	L3-L4	L4	0	5.6968	0.6835	0.8527	0	5.0725	0.6744	0.8513
	L4-L5	L5	0	6.3121	0.7266	0.9648	0	6.1705	0.789	1.0414
**P09**	Th12-L1	L1	0	3.1371	0.3553	0.4941	0	2.9389	0.3527	0.4702
	L1-L2	L2	0	3.8004	0.2718	0.3785	0	3.7606	0.3808	0.5061
**P10**	L1-L2	L2	0	2.7247	0.4278	0.5997	0	2.8764	0.4735	0.6718

Online Resource 5.
HD values of the registration process, I_1= Investigator 1; I_2= Investigator 2; HD (Hausdorff Distance, mm); RMS (Root Mean Square) min= minimum; max= maximum
