# Supplementary material for: A novel three-dimensional volumetric method to measure indirect decompression after percutaneous cement discoplasty
Source: J Orthop Translat. 2021 Apr 1;28:131–9. doi: 10.1016/j.jot.2021.02.003 (PMC8050383; doi:10.1016/j.jot.2021.02.003)
Supplement: Multimedia component 6 [file mmc6.pdf]

Patient ID	Treated segment	Cylinder height (mm)	Cylinder radius (mm)	I_1T_1			I_1T_2		
				**Subtracted cylinder volumes (preop mm³)**	**Subtracted cylinder volumes (postop mm³)**	**Δ volume (mm³)**	**Subtracted cylinder volumes (preop mm³)**	**Subtracted cylinder volumes (postop mm³)**	**Δ Volume (mm³)**
P01	L4-L5	90	11	23481.77	26791.48	3309.71	23565.42	26981.21	3415.79
	L2-L3	90	10	22440.44	24220.51	1780.07	22386	24192.35	1806.35
P02	L3-L4	90	11	25998.95	29231.33	3232.38	25916.9	29135.09	3218.19
	L4-L5	90	10	18606.19	22116.6	3510.41	18961.63	22540.93	3579.3
P03	L5-S1	90	10	10557.01	14197.34	3640.33	10654.93	14258.86	3603.93
P04	L3-L4	90	12	30949.7	33164.51	2214.81	31221.81	33359.25	2137.44
P05	L5-S1	90	11	14871.04	18819.89	3948.85	14443.92	18421.84	3977.92
P06	L1-L2	90	10	21174.4	22652.73	1478.33	21137.92	22613.81	1475.89
	L2-L3	90	10	21320.77	22826.86	1506.09	21578.23	23034.73	1456.5
P07	L3-L4	90	10	20337.3	22892.13	2554.83	20406.59	22916.85	2510.26
	L4-L5	90	10	18704.32	21819.18	3114.86	18756.52	21833.27	3076.75
P08	L3-L4	90	11	24294.87	25606.03	1311.16	24028.83	25420.71	1391.88
	L4-L5	90	12	26848.94	30482.31	3633.37	26739.6	30439.9	3700.3
P09	Th12-L1	90	10	22608.27	23847.78	1239.51	22564.76	23873.82	1309.06
	L1-L2	90	10	22436.18	23014.5	578.32	21767.42	22316.17	548.75
P10	L1-L2	90	10	24099.99	24490.95	390.96	23999.63	24401.2	401.57

Online Resource 6.
Volumetric measurements done by investigator one (I_1), at two time points (T_1, T_2)
