# Supplementary material for: A novel three-dimensional volumetric method to measure indirect decompression after percutaneous cement discoplasty
Source: J Orthop Translat. 2021 Apr 1;28:131–9. doi: 10.1016/j.jot.2021.02.003 (PMC8050383; doi:10.1016/j.jot.2021.02.003)
Supplement: Multimedia component 7 [file mmc7.pdf]

Patient ID	Treated segment	Cylinder height (mm)	Cylinder radius (mm)	I_2T_1			I_2T_2		
				Subtracted cylinder volumes (preop mm³)	Subtracted cylinder volumes (postop mm³)	Δ volume (mm³)	Subtracted cylinder volumes (preop mm³)	Subtracted cylinder volumes (postop. mm³)	Δ volume (mm³)
P01	L4-L5	90	11	23057.69	26796.4	3738.71	23099.09	26668.36	3569.27
	L2-L3	90	10	21812.04	23483.62	1671.58	22468.57	24159.33	1690.76
P02	L3-L4	90	11	25953.74	29151.15	3197.41	25939	28900.64	2961.64
	L4-L5	90	10	18677.31	22098.47	3421.16	18569.22	21943.59	3374.37
P03	L5-S1	90	10	10899.12	14372.15	3473.03	11071.42	14370.87	3299.45
P04	L3-L4	90	12	31147.28	33441.94	2294.66	31296.25	33473.92	2177.67
P05	L5-S1	90	11	14296.2	18270.97	3974.77	14222.07	17981.05	3758.98
P06	L1-L2	90	10	21032.8	22417.46	1384.66	21080.68	22418.56	1337.88
	L2-L3	90	10	21647.23	23146.92	1499.69	21777.41	23087.61	1310.2
P07	L3-L4	90	10	20548.06	22691.35	2143.29	20532.03	22715.41	2183.38
	L4-L5	90	10	18414.7	21475	3060.3	18066.85	20903.98	2837.13
P08	L3-L4	90	11	23821.27	24633.23	811.96	23806.12	24606.98	800.86
	L4-L5	90	12	26162.06	28985.67	2823.61	26883.45	30189.36	3305.91
P09	Th12-L1	90	10	22599.85	23670.88	1071.03	22750.69	23736.31	985.62
	L1-L2	90	10	22614.97	23091.49	476.52	22334.28	22890.83	556.55
P10	L1-L2	90	10	24061.54	24454.58	393.04	24025.81	24442.8	416.99

Online Resource 7.
Volumetric measurements done by the second investigator (I_2), at two time points (T_1. T_2)
