# Supplementary material for: A novel three-dimensional volumetric method to measure indirect decompression after percutaneous cement discoplasty
Source: J Orthop Translat. 2021 Apr 1;28:131–9. doi: 10.1016/j.jot.2021.02.003 (PMC8050383; doi:10.1016/j.jot.2021.02.003)
Supplement: Multimedia component 8 [file mmc8.pdf]

Patient ID	Treated segment	Cylinder height (mm^3)	Cylinder radius (mm^3)	I_3T_1			I_3T_2		
				**Subtracted cylinder volumes (preop mm^3)**	**Subtracted cylinder volumes (postop mm^3)**	**Δ Volume (mm^3)**	**Subtracted cylinder volumes (preop mm^3)**	**Subtracted cylinder volumes (postop mm^3)**	**Δ Volume (mm^3)**
P01	L4-L5	90	11	23384.98	26827.04	3442.06	22709.24	26482.48	3773.24
P02	L2-L3	90	10	22417.57	24266.97	1849.4	22146.67	23865.75	1719.08
	L3-L4	90	11	25298.15	28490.19	3192.04	25610.16	28786.74	3176.58
	L4-L5	90	10	18445.89	21894.82	3448.93	18505	22014.61	3509.61
P03	L5-S1	90	10	10842.21	14321.65	3479.44	11041.79	14307.27	3265.48
P04	L3-L4	90	12	31218.57	33304.95	2086.38	31102.72	33379.43	2276.71
P05	L5-S1	90	11	14573.66	18611.21	4037.55	14937.71	18969.46	4031.75
P06	L1-L2	90	10	21099.87	22549.31	1449.44	20932.42	22431.06	1498.64
P07	L2-L3	90	10	21349.75	22783.51	1433.76	21316.31	22818.83	1502.52
	L3-L4	90	10	20556.66	22967.03	2410.37	20349.07	22637.36	2288.29
	L4-L5	90	10	18385.93	21482.83	3096.9	18442.52	21607.34	3164.82
P08	L3-L4	90	11	24003.27	25434.62	1431.35	24005.63	25504.62	1498.99
	L4-L5	90	12	26120.15	29870.18	3750.03	26307.16	29993.66	3686.5
P09	Th12-L1	90	10	22626.94	23872.97	1246.03	22736.04	23765.07	1029.03
	L1-L2	90	10	22426.8	23045.36	618.56	22445.97	23038.85	592.88
P10	L1-L2	90	10	24097.6	24483.93	386.33	23944.13	24392	447.87

Online Resource 8.
Volumetric measurements done by investigator one (I_3), at two time points (T_1, T_2)
