# Supplementary material for: A novel three-dimensional volumetric method to measure indirect decompression after percutaneous cement discoplasty
Source: J Orthop Translat. 2021 Apr 1;28:131–9. doi: 10.1016/j.jot.2021.02.003 (PMC8050383; doi:10.1016/j.jot.2021.02.003)
Supplement: Multimedia component 9 [file mmc9.pdf]

Patient ID	Treated segment	Cylinder height (mm)	Cylinder radius (mm)	I_4T_1			I_4T_2		
				Subtracted cylinder volumes (preop mm³)	Subtracted cylinder volumes (postop mm³)	Δ Volume (mm³)	Subtracted cylinder volumes (preop mm³)	Subtracted cylinder volumes (postop mm³)	Δ Volume (mm³)
P01	L4-L5	90	11	23390.88	26885.88	3495	22700.69	26654.81	3954.12
P02	L2-L3	90	10	22419.67	24178.9	1759.23	21984.61	23885.44	1900.83
	L3-L4	90	11	25817.62	29049.93	3232.31	25996.55	29238.98	3242.43
	L4-L5	90	10	18020.35	21381.92	3361.57	18478.41	22019.51	3541.1
P03	L5-S1	90	10	11009	14477.39	3468.39	10451.22	14069.86	3618.64
P04	L3-L4	90	12	31406.05	33571.6	2165.55	30490.01	32913.3	2423.29
P05	L5-S1	90	11	15080.08	19045.2	3965.12	14577.2	18487.87	3910.67
P06	L1-L2	90	10	21089.43	22583.87	1494.44	21097.36	22596.81	1499.45
P07	L2-L3	90	10	21539.66	23016.62	1476.96	21577.02	23046.23	1469.21
	L3-L4	90	10	20118.63	22266.43	2147.8	20324.51	22503.72	2179.21
	L4-L5	90	10	18510.55	21675.99	3165.44	18463.81	21678.39	3214.58
P08	L3-L4	90	11	24299.55	25504.59	1205.04	24288.64	25478.15	1189.51
	L4-L5	90	12	26372.51	29850.95	3478.44	26755.68	30293.68	3538
P09	Th12-L1	90	10	22849.42	23796.24	946.82	22826.03	23777.02	950.99
	L1-L2	90	10	22426.15	22957.53	531.38	22357.18	22831.13	473.95
P10	L1-L2	90	10	23641.94	24168.04	526.1	24017.52	24393.35	375.83

Online Resource 9.
Volumetric measurements done by investigator one (I_4), at two time points (T_1, T_2)
