# Supplementary material for: A novel three-dimensional volumetric method to measure indirect decompression after percutaneous cement discoplasty
Source: J Orthop Translat. 2021 Apr 1;28:131–9. doi: 10.1016/j.jot.2021.02.003 (PMC8050383; doi:10.1016/j.jot.2021.02.003)
Supplement: Multimedia component 10 [file mmc10.pdf]

	preop			postop (6m FU)			Δ (preop- postop 6m FU)		
Patien ID	**ODI**	**LP**	**LBP**	**ODI**	**LP**	**LBP**	**ODI**	**LP**	**LBP**
**P01**	71.11	55	70	40.00	27	50	31.11	28	20
**P02**	68.89	60	40	66.67	60	25	2.22	0	15
**P03**	50	75	80	42.00	50	47	8.00	25	33
**P04**	66.67	60	80	40.00	50	60	26.67	10	20
**P05**	73.33	82	80	36.00	47	50	37.33	35	30
**P06**	46	60	60	22.00	60	41	24.00	0	19
**P07**	37.8	70	70	57.78	40	65	-20.00	30	5
**P08**	75.56	50	70	28.00	30	45	47.56	20	25
**P09**	75.56	75	80	32.00	70	90	43.56	5	-10
**P10**	68.89	30	80	28.00	42	80	40.89	-12	0
**Average±SD**	63.37±13.6	61.7±18	71±13	39.24±13.8	47.6±14	55±19	24.13±21.4	14±16	15.7±14

Online Resource 10.
Preop, and postop 6-month follow-up results of patient reported outcome questionnaires

.
